# Electron Paramagnetic Resonance Study of Radiation-Induced Defects in Ba_3_(PO_4_)_2_

**DOI:** 10.3390/molecules31061045

**Published:** 2026-03-20

**Authors:** Henk Vrielinck, Wouter Holvoet, Dominykas Augulis, Eliot Janssens, David Van der Heggen, Dirk Poelman

**Affiliations:** 1 Department of Solid State Sciences, Ghent University, Krijgslaan 285-S1, B-9000 Gent, Belgium; 2 Institute of Photonics and Nanotechnology, Faculty of Physics, Vilnius University, Sauletekio al. 3, LT-10257 Vilnius, Lithuania

**Keywords:** dosimetry, radio-photoluminescence, Eu-doped Ba_3_(PO_4_)_2_, EPR, radiation defects

## Abstract

We report an electron paramagnetic resonance (EPR) study of radiation-induced defects in Ba_3_(PO_4_)_2_, aiming to understand their role in radio-photoluminescence (RPL). Ba_3_(PO_4_)_2_ is a promising host for rare-earth dopants in optical and dosimetric applications. We compare the effects of ultraviolet (UV) and X-ray irradiation on the electron trapping by Eu^3+^, as well as the formation of intrinsic defects by radiation in Eu-doped and undoped samples. Both irradiation types generate Eu^2+^ centers with axial symmetry at one specific Ba lattice site, as confirmed by Q-band EPR. Additional EPR signals near g≈2 reveal radiation-induced centers unrelated to Eu dopants. Detailed analysis of X- and Q-band spectra identifies an H^0^ center and two electron-trapping defects, one tentatively assigned to an oxygen vacancy (F-type center). These findings pave the way for understanding the complex defect landscape governing charge trapping and stability in Ba_3_(PO_4_)_2_.

## 1. Introduction

Ba_3_(PO_4_)_2_ is a wide-bandgap crystalline compound characterized by high chemical and thermal stability. In powder form it is traditionally synthesized via high-temperature solid-state diffusion [1,2,3,4], although wet-chemical routes have also been reported [5,6,7]. Recently, its low toxicity and the cost-effectiveness of wet-chemical synthesis have inspired a broad range of applications, including use as an additive in lubricants [5], as a photocatalyst [6], and as an adsorbent for water purification, in particular to eliminate methyl blue [7]. More conventionally, Ba_3_(PO_4_)_2_ serves as a host lattice for luminescent transition-metal and rare-earth (RE) ions in diverse optical applications, particularly as a down-conversion phosphor for lighting [2,3,4,8,9,10]. For most di- and trivalent dopants, substitution occurs on Ba^2+^ sites, whereas pentavalent transition-metal ions, like Mn^5+^, can be stabilized in the P^5+^ position [11]. Mn^5+^ has recently attracted considerable attention due to its near-infrared (NIR)-II luminescence (1000–1350 nm) under NIR-I excitation (650–950 nm), making it highly relevant for in vivo luminescence imaging. Furthermore, in vivo luminescence-based thermometry has been demonstrated for Sr_3_(PO_4_)_2_:Mn^5+^ and Ba_3_(PO_4_)_2_:Mn^5+^ [12].

Our primary interest in Ba_3_(PO_4_)_2_ lies in luminescence-based imaging and dosimetry of ionizing radiation. Tale et al. investigated the thermally stimulated luminescence (TSL) of Eu^2+^-activated and undoped Ba_3_(PO_4_)_2_ X-ray phosphor screens following X-ray excitation at liquid-nitrogen temperature [13]. Schipper et al. demonstrated that the X-ray storage capacity of Eu^2+^-activated Ba_3_(PO_4_)_2_ is significantly enhanced by co-doping with La^3+^, Y^3+^, and other trivalent lanthanides [2,14]. They examined both the TSL and the optically stimulated luminescence (OSL) of these phosphors. We recently showed that Ba_3_(PO_4_)_2_ doped with Sm^3+^, Eu^3+^, and Yb^3+^ exhibits radiation-induced changes in RE-related luminescence upon UV exposure, associated with valence state conversion of the dopant to 2+ [15]. This phenomenon, known as radio-photoluminescence (RPL), is particularly promising in Eu-doped Ba_3_(PO_4_)_2_ because its RPL charging spectrum aligns with the erythemal action spectrum [15]. Moreover, simultaneous excitation of Eu^2+^ and Eu^3+^ photoluminescence in the UV enables dose assessment via the intensity ratio of violet Eu^2+^ and red Eu^3+^ emission. The present work reports on the extension of these RPL studies to higher-energy photons, notably X-rays.

TSL, OSL, and RPL in inorganic phosphors all rely on stable trapping of charge carriers generated by irradiation [16,17]. TSL and OSL involve recombination luminescence, which erases stored dose information during readout. In contrast, RPL excitation does not induce carrier detrapping, thereby preserving dose information—a key advantage of this mechanism [17,18]. To date, only a few RPL phosphors have achieved practical use, notably LiF [19], Al_2_O_3_:C,Mg [20] and Ag-doped phosphate glasses [21]. Over the past decade, Sm^3+^- and Eu^3+^-doped crystalline and glassy materials have emerged as promising RPL phosphors [18]. Their readout relies on electron trapping by Sm^3+^ or Eu^3+^, although hole traps and alternative electron traps critically influence RPL stability and efficiency. These traps also govern the TSL and OSL behavior. The present work addresses radiation defects in Eu-doped Ba_3_(PO_4_)_2_ and their impact on RPL performance.

For effective RPL, Eu in Ba_3_(PO_4_)_2_ should be predominantly in the 3+ state prior to irradiation. In this way the change in luminescence intensity ratio or color upon exposure to X-rays is maximized, increasing the RPL sensitivity. This is achieved by a final sintering step in the solid-state synthesis, at high temperature in an oxidizing atmosphere [15]. Under ∼400 nm near-UV excitation, these samples exhibit line emission in the orange–red region, with the strongest peak at 615 nm, characteristic of the intra 4f^6^ configuration transitions of Eu^3+^. The occurrence of not one but two ^5^D_0_ → ^7^F_0_ transitions near 580 nm indicates that Eu^3+^ occupies at least two sites in Ba_3_(PO_4_)_2_. At shorter excitation wavelengths, down to 350 nm, no indications of broad band Eu^2+^ emission are observed. However, exposure to UV light with λ<320 nm generates a broad, Eu^2+^-related emission band peaking around 420 nm [15], with an excitation spectrum spanning the range λ∼ 300–380 nm. A valence state change in Eu thus occurs from photon energies of about 4 eV, way below the band gap energy of Ba_3_(PO_4_)_2_, which is estimated at 8.2 eV [4]. Electron paramagnetic resonance (EPR) spectroscopy showed that the Eu^2+^ center that is formed after electron trapping resides at a single site with axial symmetry [15]; although in samples sintered in a reducing atmosphere, Eu^2+^ occupies both Ba sites with trigonal axial symmetry in the rhombohedral Ba_3_(PO_4_)_2_ lattice [1]. Furthermore, for X-ray exposure of Ba_3_(PO_4_)_2_, Tale et al. [13] showed that the sintering atmosphere strongly affects glow curves and thus defect structures, while Schipper et al. [2,14] reported that RE^3+^ incorporation also modifies radiation-induced charge trapping behavior, sometimes dopant-specific, although Y^3+^ and La^3+^ mainly influence intrinsic defects.

In this paper we aim to clarify the role of radiation-induced defects in the RPL process in Ba_3_(PO_4_)_2_:Eu^3+^.

## 2. Results and Discussion

### 2.1. X-Ray Induced RPL and Eu^2+^-Related Centers in Eu-Doped Ba_3_(PO_4_)_2_

#### 2.1.1. Results

**Figure 1:**
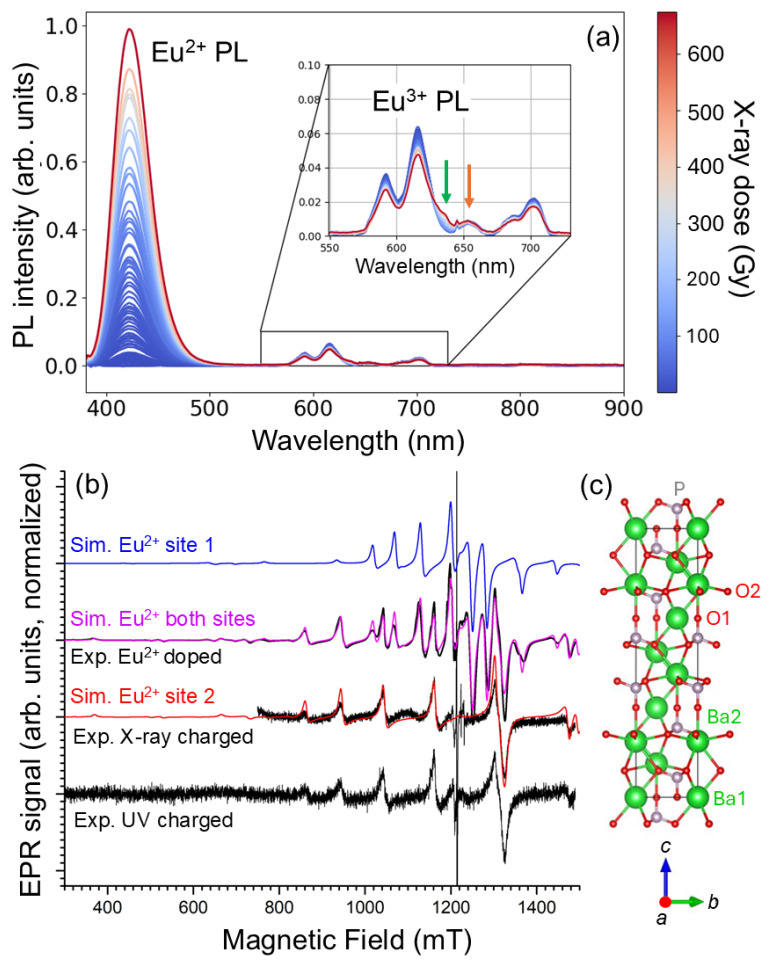
(**a**) PL spectrum under 375 nm LED excitation of Ba_3_(PO_4_)_2_ as a function of the X-ray radiation dose. The inset shows a zoom-in on the Eu^3+^-related intra $4f^{6}$ configuration emission, where the luminescence intensity decreases with radiation dose. Around 640 nm (green arrow) a band grows in, while the intensity of a band at 655 nm (orange arrow) is hardly affected by irradiation. (**b**) Q-band EPR spectra ($f_{\mathrm{MW}}=34.000$ GHz) of Ba_3_(PO_4_)_2_; bottom trace—black: Eu^3+^-doped after UV charging (λ=265 nm, ∼$1J/{\mathrm{cm}}^{-2}$); 2nd trace—black: Eu^3+^-doped after X-ray charging, red: simulation for Eu^2+^ Site 2 (simulation parameters in Table 1); 3rd trace—black: Eu^2+^-doped, magenta: simulation for both Eu^2+^ sites; top trace—blue: simulation for Eu^2+^ Site 1. (**c**) Unit cell of Ba_3_(PO_4_)_2_ with labeling of the crystallographic positions.

We previously established that λ≈ 275 nm UV irradiation of Ba_3_(PO_4_)_2_:Eu^3+^ produces Eu^2+^ centers, resulting in violet photoluminescence and an EPR spectrum of a single center with spin S = 7/2 and axial symmetry [15]. Figure 1 demonstrates that X-ray irradiation also leads to partial photoreduction in the Eu^3+^ dopant. Figure 1a shows how the violet Eu^2+^ $4f^{6}5d^{1}\to 4f^{7}$ band grows with an increasing X-ray dose. Meanwhile, the intensity of the Eu^3+^ intra $4f^{6}$ configuration emission decreases, as is clearly seen in the inset of the figure. Around 640 nm a shoulder on the main emission line in this region grows in, and a band at 660 nm seems only very slightly affected by irradiation. The origin of these luminescence features is not clear at this moment, but they may be related to the intrinsic defects.

Figure 1b compares the experimental EPR spectra after UV and X-ray charging of Ba_3_(PO_4_)_2_:Eu^3+^ with the EPR spectrum of Eu^2+^-doped Ba_3_(PO_4_)_2_, obtained by sintering the sample in reducing atmosphere. Except for narrow transitions in the magnetic field range near g=2, which are discussed further on, the spectra of the UV and X-ray charged Eu^3+^-doped samples are identical. All broad EPR transitions in the spectra are attributed to a single Eu^2+^ center with axial symmetry, as shown in the simulation, using the g and zero-field splitting parameters earlier reported [15] and listed in Table 1 (Eu^2+^ Site 2). The simulations are performed by diagonalizing the spin Hamiltonian ($\mu_{B}$ represents the Bohr magneton).

(1) $$\begin{array}{cc} \hat{H} & =g\mu_{B}\vec{B}\cdot \hat{\vec{S}}+B_{2}^{0}\left(3{\hat{S}}_{z}^{2}-S\left(S+1\right)I\right)\\ \; & +B_{4}^{0}\left[\left(35{\hat{S}}_{z}^{4}-\left(30S\left(S+1\right)-25{\hat{S}}_{z}^{2}\right)+\left(3S^{2}{\left(S+1\right)}^{2}-6S\left(S+1\right)\right)I\right)\right.\\ \; & \left.\;\;+20\sqrt{2}\left({\hat{S}}_{z}\left({\hat{S}}_{+}^{3}+{\hat{S}}_{-}^{3}\right)+\left({\hat{S}}_{+}^{3}+{\hat{S}}_{-}^{3}\right){\hat{S}}_{z}\right)\right] \end{array}$$

The first term on the right-hand side represents the electronic Zeeman interaction between the magnetic field $\vec{B}$ and the electron spin. The second and third terms represent the second and fourth-rank zero-field splitting (ZFS), respectively.

In order to avoid overfitting of the broad powder spectra that extend over a wide field range, we assume (for both Eu^2+^ centers) the g tensor to be isotropic and ignore the sixth-rank ZFS contribution. Furthermore, we assume that the fourth-rank ZFS Hamiltonian preserves cubic symmetry, and thus introduces only one parameter, $B_{4}^{0}$. The second-rank ZFS parameter $B_{2}^{0}$ reflects the deviation of the center from cubic symmetry.

The broad transitions for Eu^2+^ Site 2, observed in the Eu^3+^-doped Ba_3_(PO_4_)_2_ after irradiation with X-rays or UV, also appear in the spectrum of Eu^2+^-doped Ba_3_(PO_4_)_2_ (synthesized under reducing atmosphere), but additional broad lines occur in this spectrum. In Ref. [15] we established that these are due to a second Eu^2+^ center with axial symmetry with a smaller $B_{2}^{0}$ parameter (Eu^2+^ Site 1). The EPR spectrum simulation for the reduced sample is a linear combination of the simulations for both Eu^2+^ sites in a (Site 1:Site 2) occupancy ratio (3:4). Even though experimental spectra are fairly well reproduced, the simulations indicate that for perfect reproduction, a distribution in the zero field splitting parameters needs to be taken into account. Therefore, the 3:4 ratio should only be regarded as indicative: the concentration of Eu^2+^ in the two sites is comparable.

#### 2.1.2. Discussion

The occurrence of two Eu^2+^ centers in the sample sintered in reducing atmosphere is readily understood when inspecting the crystal structure of Ba_3_(PO_4_)_2_ [1]. There are indeed two crystallographic sites for Ba^2+^ in this structure (see Figure 1c). Both have axial site symmetry, so at first sight, ambiguity remains in the site assignation of the two Eu^2+^ sites. However, one of the two sites, Ba1, is coordinated by 12 O^2−^ neighbors. This coordination is derived from a close-packed cubic coordination by a fairly large axial distortion along a <111> axis of the cube (c-axis of the crystal), preserving inversion symmetry. The tenfold O^2−^ coordination of the Ba2 site strongly differs from a cubic environment, and lacks inversion symmetry. Considering the geometry of the undistorted Ba lattice sites, it is thus very plausible that the paramagnetic Eu^2+^ Site 1 center, with the smaller $B_{2}^{0}$ parameter, corresponds to Eu^2+^ substitution on the Ba1 lattice position, and Eu^2+^ Site 2 occupies the Ba2 position. The Ba2 site is twice as abundant as Ba1 in the Ba_3_(PO_4_)_2_ lattice. Considering the uncertainty in the relative EPR spectral intensities for the two Eu^2+^ centers (3:4) mentioned earlier, EPR provides no clear indication that Eu^2+^ exhibits a preference for occupying a particular Ba site.

In UV or X-ray irradiated Eu^3+^ doped Ba_3_(PO_4_)_2_ we did not observe the Eu^2+^ Site 1 center. Simulations indicate that the intensity for this center in the spectrum after UV or X-ray irradiation is at least eight times lower than that for Eu^2+^ Site 2 (see Supplementary Information, Figure S1). Eu^2+^ production by irradiation thus exhibits a clear preference for Site 2.

One possible explanation for this selectivity is that Eu^3+^ incorporation in the lattice is site selective; this means that the small Eu^3+^ ion exhibits a clear preference for substitution on the Ba2 position. However, both in Ref. [4] and in Ref. [15], two distinct Eu^3+^ sites have been observed in the PL spectra. Lazoryak et al. [4] even explicitly link this to incorporation of Eu^3+^ on the Ba1 and Ba2 sites. If there is no clear site preference for Eu^3+^ incorporation, then the EPR results imply that stable electron trapping at Eu^3+^ is site-selective.

It should be noted that, besides Eu^3+^ substitution on the two Ba sites in the lattice, other explanations exist for the occurrence of two distinct Eu^3+^ luminescence centers in Ba_3_(PO_4_)_2_. Indeed, Eu^3+^ substitution for Ba^2+^ requires charge compensation, and the observed two Eu^3+^ centers might differ in this respect, rather than in their Ba^2+^ lattice position. Moreover, it has been shown that Ce^3+^ and Tb^3+^ doping in Ba_3_(PO_4_)_2_ at concentrations higher than 0.3 mol% leads to the formation of a RE^3+^-concentrated crystal phase (Ba_3_RE(PO_4_)_3_) [22]. The PL of Eu^3+^ in this concentrated phase (earlier reported in [23]), or in regions of high local concentration in the Ba_3_(PO_4_)_2_ lattice, might also be distinguishable from that of isolated Eu^3+^ in a regular Ba^2+^ site of Ba_3_(PO_4_)_2_. We found no evidence for a Eu-concentrated crystal phase in the XRD patterns of our samples, only weak traces of Ba_5_(PO_4_)_3_(OH) [24] in some of the in-house synthesized samples and a somewhat higher concentration of this Ba-hydroxyapatite phase in commercial undoped Ba_3_(PO_4_)_2_ (see Supplementary Information, Figure S2).

We are currently further investigating the origin of the observed site selectivity for Eu^2+^ production by irradiation, preferential incorporation of Eu^3+^ or preferential electron trapping, by studying the EPR spectra of paramagnetic RE^3+^ ions in Ba_3_(PO_4_)_2_, in particular Gd^3+^.

### 2.2. Other Radiation-Induced Centers in Eu-Doped Ba_3_(PO_4_)_2_

#### 2.2.1. Results

**Figure 2:**
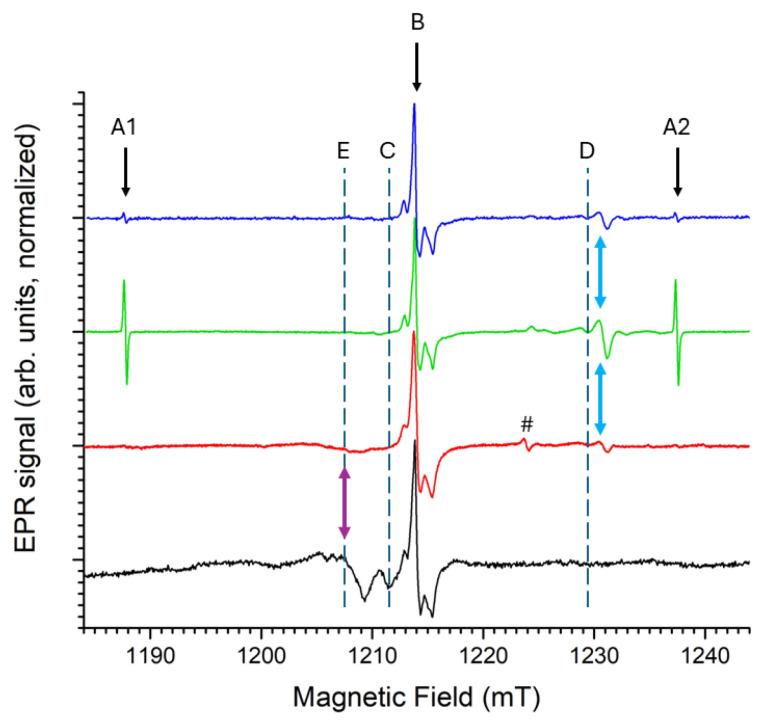
Zoom of the EPR spectra in Figure 1b. Black: UV charged (λ=265 nm, $∼1J/{\mathrm{cm}}^{-2}$) Eu-doped; red: X-ray irradiated Eu-doped (450 Gy); green: commercial undoped Ba_3_(PO_4_)_2_ ∼90 min after 225 Gy X-ray irradiation; blue: same sample as green, ∼70 h later. Spectra are normalized to the maximum of the signal labeled as “B”. Signal labels and markings are explained in the text.

In the magnetic field region near g = 2 (≈1214 mT at 34 GHz) the EPR spectra of UV and X-ray charged Ba_3_(PO_4_)_2_:Eu^3+^ exhibit narrow EPR lines that are not directly related to Eu^2+^. Figure 2 shows a zoom of the spectra shown in Figure 1 on this region. The EPR spectrum of X-ray irradiated commercial, undoped Ba_3_(PO_4_)_2_ is added for comparison. The latter spectrum is shown, recorded directly after irradiation, and about 72 h after irradiation, keeping the sample under ambient conditions.

Schipper et al. [2,14] studied paramagnetic radiation defects in Ba_3_(PO_4_)_2_ doped with various RE^3+^ ions. They found that the radiation-induced EPR spectrum strongly depends on the dopant and on the sintering atmosphere and duration. In Ref. [2] they labeled five EPR components (A–E) in the spectra of irradiated La^3+^-doped Ba_3_(PO_4_)_2_. Component A is a doublet split by about 50 mT. We observe this or a similar doublet in the undoped commercial sample (A1 and A2 in Figure 2), but not, or only very weakly, in the Eu^3+^-doped samples. We also did not observe it in other in-house synthesized samples. Moreover, this signal has decayed by a factor of ∼10 after 3 days under ambient conditions. The analysis and discussion of this paramagnetic center are given in Section 2.3.

For the other four components, Schipper et al. provide g-factors: the field positions corresponding to these g-factors are marked in Figure 2. It is clear that we observe the same, or at least very similar, radiation-induced paramagnetic centers in the in-house synthesized Eu-doped and the commercial undoped sample we studied. These paramagnetic radiation centers should thus be intrinsic or related to very common impurities in Ba_3_(PO_4_)_2_.

All spectra exhibit a B-signal at g=2.001. In both samples it is the most stable non-Eu^2+^-related center produced by irradiation. Relative to the Eu^2+^ EPR signal, it is much weaker after UV than after X-ray irradiation. We discuss this spectral component in Section 2.5. At g=1.976 (D signal observed by Schipper et al.) no or only a very weak EPR line is seen in the spectra, but at a slightly higher magnetic field, in the spectra produced by X-ray irradiation, an asymmetric EPR line is clearly seen (blue arrows). There is little doubt that this is the component observed by Schipper et al., and we will therefore label it here as D. Its EPR characteristics in undoped Ba_3_(PO_4_)_2_ and identification are further discussed in Section 2.4. At room temperature, signal D decays faster than B, but it is more stable than A. It should be noted that in spite of their considerable amplitude, in UV or X-ray irradiated Eu-doped Ba_3_(PO_4_)_2_, signals B and D represent a small total integrated intensity in comparison to the very broad signal of Eu^2+^. Via spectral simulations we estimate that a short time after X-ray irradiation, the total integrated intensity of the Eu^2+^-related EPR component is two to three orders of magnitude larger than that of the B and D signals, which have comparable intensities.

At the g-position of component C (g=2.005, according to Schipper et al. [2]), we only observe a small EPR line in the UV irradiated Eu-doped sample. Around g=2.012, the *g*-factor for signal E of Schipper et al., we do observe an EPR signal in the Eu-doped sample. Its intensity is much weaker in the undoped commercial sample. Schipper et al. observe a four-line hyperfine structure for this component, which is absent in our spectra. On the contrary, the EPR signal we detect in this field region has a poorly resolved structure, which seems to be different for UV and X-ray irradiation. The stability of these signals at room temperature is comparable to that of signal B. Since we observe them most clearly in the presence of large concentrations of trapped electron centers (Eu^2+^), it is tempting to assign these signals to trapped hole centers. Hole trapping in Ba_3_(PO_4_)_2_ is expected to occur at or near the (PO_4_)^3−^ anion units. Tale et al. [13] observed that the intrinsic trapped hole centers at (PO_4_)^3−^ decay around 130 K, giving rise to an intense TSL signal. If they are indeed trapped hole centers, the room temperature stable C and E centers should thus be defect or impurity-related. The lack of structure at room temperature makes a further analysis of the C and E EPR signals difficult at this point. Therefore, we do not discuss these signals further in this paper.

#### 2.2.2. Discussion

It is not a priori evident that UV and X-ray irradiation would produce the same type of defects. Indeed, photoreduction in Eu in Ba_3_(PO_4_)_2_: Eu^3+^ under UV exposure was observed between 3.9 eV $<E_{\mathrm{phot}}<$ 6.2 eV [15]. Lazaryak et al. [4] identified the exciton band and onset for valence to conduction band transitions in Eu-doped Ba_3_(PO_4_)_2_ at 7.5 eV and about 8.2 eV, respectively. The UV light used for charging has considerably lower photon energy, so it is safe to assume that electron–hole pair production in the host is excluded. As we explained earlier [15], a local charge transfer, e.g., from ligand to RE metal, is responsible for the reduction in Eu^3+^ in the UV charging experiments. X-rays with photon energies in the keV range do produce free electron–hole pairs, so that valence state changes can be expected for all stable electron and hole traps in the material. Indeed, we observe clear differences between EPR spectra after UV and X-ray charging. Relative to the Eu^2+^ signal we observe a much smaller intensity of the B signal, and no D signal (above the detection threshold) for charging with UV (see Supporting Information, Figure S3). The signal marked with # in the spectrum of X-ray irradiated Ba_3_(PO_4_)_2_:Eu^3+^, occurring at g≈1.985 and not mentioned by Schipper et al. [2,14], is also not detected after UV charging.

In the remainder of the results section, the analysis of the three most prominent radiation-induced EPR signals in undoped Ba_3_(PO_4_)_2_ (A, B and D) are presented. X and Q-band EPR are combined for a consistent interpretation. All these centers have electron spin S = 1/2. The spin Hamiltonian to diagonalize for their analysis consists of an electron Zeeman term and terms for hyperfine (and nuclear Zeeman) interactions with the central or neighboring nuclei.

(2) $$\begin{array}{c} \hat{H}=g\mu_{B}\vec{B}\cdot \hat{\vec{S}}+\sum_{i}\left(\hat{\vec{S}}\cdot {\overset{↔}{A}}_{i}\cdot {\hat{\vec{I}}}_{i}-g_{N,i}\mu_{B}\vec{B}\cdot {\hat{\vec{I}}}_{i}\right) \end{array}$$

Schipper et al. [2,14] only identified signal A as due to atomic hydrogen H^0^ centers, but did not propose models or suggest an origin for the other centers.

### 2.3. Signal A: H^0^ Centers

#### 2.3.1. Results

**Figure 3:**
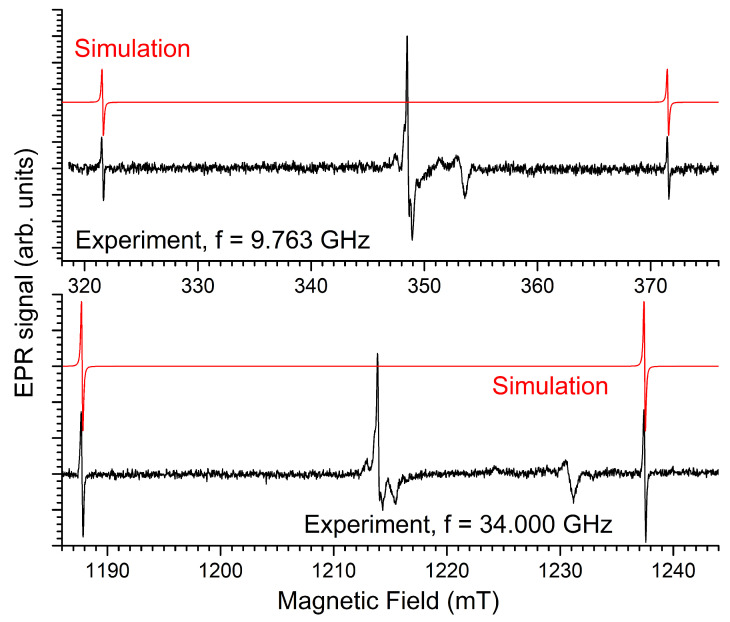
X- and Q-band EPR spectra of commercial, undoped Ba_3_(PO_4_)_2_, after X-ray irradiation at room temperature (40 kV, 40 mA, 900 s, ∼225 Gy), recorded at room temperature. Microwave power: 1 mW. Field modulation: 0.1 mT amplitude at 100 kHz. Black traces are experimental spectra; simulations using the parameters in Table 2 are shown in red.

Figure 3 shows the X- and Q-band EPR spectra of undoped Ba_3_(PO_4_)_2_, recorded within the first hour after irradiation, with small modulation amplitude (0.1 mT) in order to avoid overmodulation of the narrow lines of this doublet EPR signal. Simulations are also shown, using the same g, hyperfine and linewidth parameters (Table 2) for the two bands. For comparison, the *g* and A parameters published in Ref. [2] are also presented.

#### 2.3.2. Discussion

At first glance, the H^0^ signal we observe in the undoped Ba_3_(PO_4_)_2_ differs significantly from that reported in Ref. [2]. The discrepancy is largest for the g-factor, but the difference can easily be explained. Schipper et al. state that the “(mean) g value of the signal is 2.013” [2], which may imply that it was determined from the mean field value of the two hyperfine lines. Since the hyperfine interaction is large, there is a considerable second-order hyperfine correction to the resonance field positions $B_{\mathrm{res}}\left(M_{I}\right)$ (for ^1^H, I = 1/2 and $M_{I}=\pm 1/2$):

(3) $$\begin{array}{c} B_{\mathrm{res}}\left(M_{I}\right)=\frac{hf}{g\mu_{B}}-AM_{I}-\frac{g\mu_{B}}{4hf}A^{2}. \end{array}$$

This second-order downshift of the resonance magnetic fields, the third term on the right-hand side of Equation (3), is proportional to the square of the hyperfine strength A, and inversely proportional to the microwave frequency f. It amounts to ≈1.8 mT for the X-band spectrum, and for Q-band ≈0.5 mT. Ignoring this second-order correction would shift the g-factor determined from the X-band spectrum to 2.0127, in close agreement with the result in Ref. [2].

The observed hyperfine splitting is somewhat smaller than earlier reported [2], which has no evident explanation. No error was stated for the literature hyperfine splitting. Still, a difference of 0.6 mT, about 1% of the measured hyperfine splitting value, is well measurable in EPR spectroscopy. It can therefore not be excluded that the H^0^ centers produced by X-rays in commercial Ba_3_(PO_4_)_2_ differ slightly from those reported for RE^3+^-doped Ba_3_(PO_4_)_2_, which were synthesized and sintered in reducing atmosphere [2].

The H^0^ centers are generated by the trapping of radiation-produced electrons by H^+^ [2]. It was proposed in Ref. [25] that H^+^ can be weakly bound to the (PO_4_)^3−^ anions, and that this would lead to electron trapping that is stable at room temperature. Schipper et al. [2] suggested that H^+^ bound to (PO_4_)^3−^ next to a Ba^2+^ vacancy would function as a charge compensator for the RE^3+^ ion substitution for Ba^2+^. However, they did not systematically observe radiation-induced production of H^0^ in all RE^3+^-doped Ba_3_(PO_4_)_2_ phosphors studied [14]. We observed no or only weak traces of H^0^ in the in-house synthesized RE^3+^-doped samples sintered under air or reducing atmosphere. The final sintering in our synthesis procedure occurred at slightly higher temperatures than in the earlier study (1300 °C vs. 1200 °C), which may explain this observation. In line with this reasoning, Schipper et al. [14] observed that in most RE^3+^-doped samples (La^3+^-doping being an exception), prolonged sintering (15 h instead of 8 h) strongly suppressed H^0^ produced upon irradiation.

### 2.4. Signal D at g≈1.976

#### 2.4.1. Results

**Figure 4:**
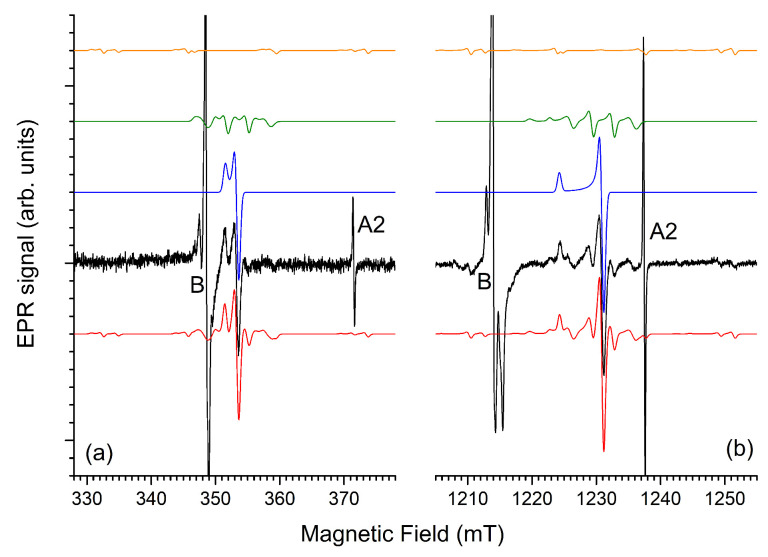
(**a**) X-band (9.763 GHz) and (**b**) Q-band (34.000 GHz) EPR spectra of Ba_3_(PO_4_)_2_ short time (∼1.5 h) after X-ray irradiation (∼225 Gy). Black traces are experimental spectra, recorded with 0.3 mT modulation amplitude. Colored traces are simulations for signal D with spin Hamiltonian parameters in Table 3: Blue: without hyperfine interactions; green: hyperfine interaction with a shell of 3 equivalent Ba nuclei; orange: larger hyperfine interaction with one Ba nucleus; red: sum of the three contributions. The simulations assume a residual isotropic Gaussian peak-to-peak linewidth of 0.6 mT. Contributions of signals A (line A2) and B are marked in the spectra as well.

The asymmetric signal occurring in Figure 3 at about 1230 mT in the Q-band, i.e., at fields just above g=1.976, has a considerably larger linewidth than the H^0^ doublet. This spectral component is therefore better observed at higher modulation amplitude, as shown in Figure 4.

**Table 3:** Spin Hamiltonian parameters for center D (g≈1.976) in Ba_3_(PO_4_)_2_. The literature parameters for F centers in BaFCl [26,27] and for F^+^ in BaO [28] are included for comparison. Hyperfine parameters refer to ^137^Ba; values for ^135^Ba are obtained by scaling with the nuclear $g_{N}$ ratio. Numbers in braces indicate the number of equivalent nuclei.

Parameter	Comp.	D Center	F(F^−^)	F(Cl^−^)	F^+^
		Ba_3_(PO_4_)_2_	BaFCl	BaFCl	BaO
*g*	⊥	1.9733±0.0005	1.9695	1.9798	1.936
	‖	1.9842±0.0005	1.9836	1.9690	1.936
*A*(^137^Ba^*A*^) (mT)	*x*	13.5±0.2 {1}	7.72 {4}	3.53 {1}	6.8 {6}
	*y*	13.5±0.2 {1}	7.72 {4}	3.53 {1}	6.8 {6}
	*z*	13.5±0.2 {1}	7.72 {4}	4.86 {1}	6.8 {6}
*A*(^137^Ba^*B*^) (mT)	*x*	3.35±0.10 {3}		3.53 {4}	
	*y*	3.35±0.10 {3}		3.48 {4}	
	*z*	3.15±0.10 {3}		4.16 {4}	
A-strain(^137^Ba^*B*^) (mT)	x,y	0.6±0.2			
	*z*	0.1±0.2			
Linewidth (Gaussian, mT)		0.6±0.1			

The asymmetric shape of the line suggests axial g-anisotropy. Simulations in Figure 4 confirm this (blue traces). The higher modulation amplitude also reveals the side-structure from hyperfine interactions with nuclei with limited natural abundance. The Ba host nuclei, which have two natural magnetic isotopes, both with I = 3/2 (^135^Ba, $g_{N}=0.55863$, 6.592%; ^137^Ba, $g_{N}=0.62491$, 11.232%) [29], are most likely responsible for this interaction. The experimental X- and Q-band spectra are properly reproduced when assuming a very strong interaction with a single Ba nucleus (Ba^A^) and a weaker interaction with a shell of three equivalent Ba nuclei (Ba^B^). The simulations (spin Hamiltonian parameters in Table 3) show the contributions of these interactions separately, as well as an isotope abundance-weighted sum of these contributions, matching the experimental spectra. In this simulation approach we neglect the contribution of centers with more than one magnetic Ba nucleus within this set of four Ba neighbors (Ba^A^ and Ba^B^).

For Ba^A^, we assumed the interaction to be isotropic: only the splitting of the $g_{⊥}$ spectral component was observed in the spectra, while that of the parallel feature remained below the noise level. The hyperfine structure from the three Ba^B^ nuclei is resolved on the $g_{⊥}$ and on the $g_{\|}$ features of the spectrum and exhibits a small anisotropy. Moreover, in order to obtain good agreement with the experimental spectrum, a strain in the $A_{⊥}$ parameter is taken into account, very probably related to a rhombicity of the A tensor and non-coincidence of its principal axes with those of the defect’s g tensor. Indeed, the only restriction imposed by symmetry on this A tensor is that one principal direction lies in the $g_{⊥}$ plane.

The observed hyperfine interactions are dominated by the isotropic contribution and seem large considering the small $g_{N}$ factors of the natural Ba magnetic isotopes. The unpaired electron density on the neighboring nuclei can be estimated by comparing the observed isotropic hyperfine interactions with the hyperfine field expected for 100% spin localization in the Ba 6s orbital. Using the data from Ref. [30], the latter hyperfine field for the ^137^Ba isotope is calculated to be 218 mT. The spin delocalization onto Ba^A^ would thus be estimated as 6% and 1.5% on each of the three nuclei in the shell Ba^B^.

#### 2.4.2. Discussion

The F center in halides, i.e., an electron trapped at a halide vacancy, and its analogon in oxides, the F^+^ center, a single electron trapped at an oxide vacancy, are characterized by strong delocalization of the unpaired electron density onto the first cation coordination shell. In Table 3 the g and hyperfine parameters of the D center are compared with those for the two F centers (electron trapped in a F^−^ or in a Cl^−^ vacancy) in BaFCl [26,27]. The magnitude and anisotropy of the shifts of the principal g values from the free-electron value, as well as the total delocalization onto the first cation coordination shell for these F centers agree very well with our observations for this radiation center in Ba_3_(PO_4_)_2_. Furthermore, a large negative g shift from the free electron value and large Ba hyperfine splitting have also been observed for the cubic F^+^ center in BaO ([28], see Table 3). Therefore, we propose an F^+^ center as the model for center D.

**Figure 5:**
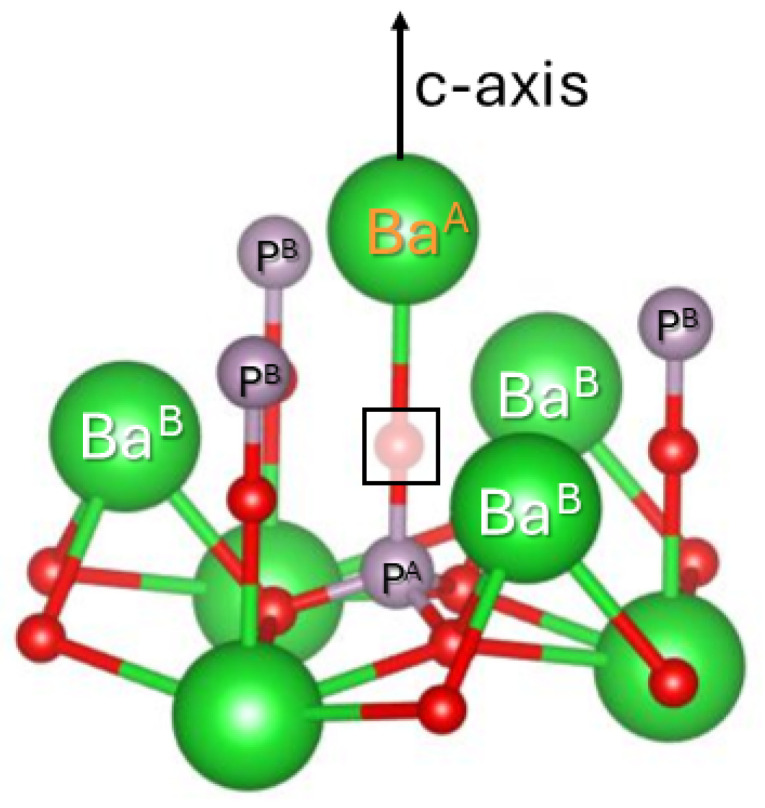
Nearest environment of a vacancy at the O1 position in the Ba_3_(PO_4_)_2_ lattice. Oxygen: red; phosphorus: grey; barium: green.

The axial g tensor symmetry of the center is preserved when lowering the sample temperature down to 85 K (see Supporting Information, Figure S4). This allows us to identify the crystallographic position of the oxygen vacancy as O1. Figure 5 shows the nearest environment of an oxygen vacancy at this position. The nearest Ba^2+^ ions belong to two shells: one ion (Ba^A^) along the c-axis connecting this oxygen position to this ion at a distance of R = 0.264 nm, and a shell of three ions (Ba^B^) at R = 0.324 nm in a plane perpendicular to the c-axis. This explains the observed hyperfine structure.

For the Ba nuclei at R = 0.324 nm, the point-dipole contribution $A_{\mathrm{dip}}$ to the anisotropy of the hyperfine splitting

(4) $$\begin{array}{c} 3A_{\mathrm{dip}}=A_{\|}-A_{⊥}=3\frac{\mu_{0}}{4\pi }\cdot \frac{g_{N}\mu_{N}}{R^{3}} \end{array}$$

is 0.028 mT. The large residual linewidth of this spectrum and the large strain in $A_{⊥}$ obscure the true anisotropy of this hyperfine tensor. Still, the anisotropy resolved in the experiment is already larger than the point-dipole contribution. Hence, the unpaired electron density appears to be delocalized onto neighboring Ba p orbitals as well.

In the pristine lattice O1 also has four ^31^P nuclei in its nearest environment, one at R = 0.154 nm (P^A^ in Figure 5) and three equivalent at R = 0.360 nm (P^B^). It is therefore surprising that no hyperfine interactions with ^31^P nuclei (I = 1/2, 100% abundance, g_*N*_ = 2.2632 [29]) are resolved in the spectra, in particular the one with the nearest nucleus: P^A^. Its point-dipole contribution $A_{\mathrm{dip}}$ = 0.31 mT may remain unresolved in the residual linewidth of the spectrum. However, a delocalization of the unpaired electron density of only 1% onto this nucleus would lead to a hyperfine interaction of 5.7 mT. A splitting of this magnitude is clearly not observed in the experimental spectrum. As an explanation for this absence of ^31^P hyperfine splitting, we may hypothesize that the nearest P to the O vacancy site is replaced by an impurity with low natural abundance in magnetic isotopes, like C, Si or S. Higher resolution hyperfine techniques, like electron nuclear double resonance (ENDOR) or pulsed EPR techniques, are required to test the validity of such a hypothesis experimentally. First principle modeling may also help in explaining this puzzling absence of resolved ^31^P hyperfine interaction.

### 2.5. Signal B at g ≈ 2.001

#### 2.5.1. Results

**Figure 6:**
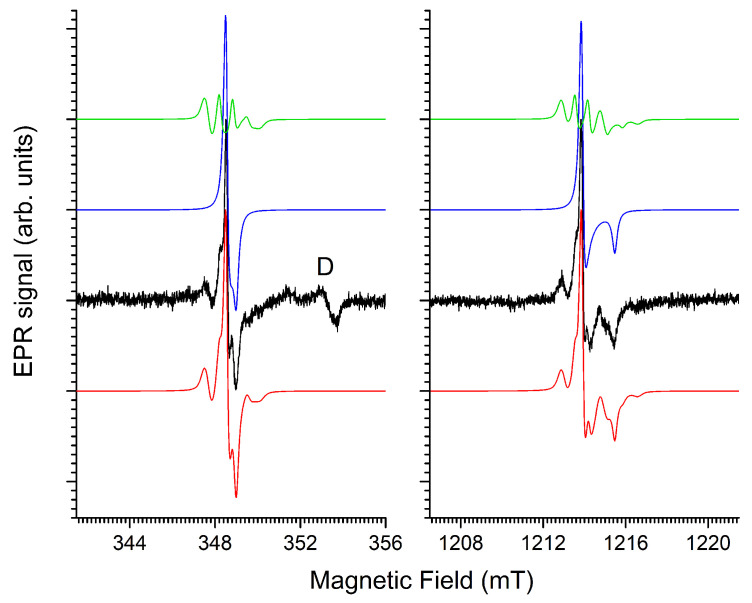
EPR signal of X-ray irradiated undoped Ba_3_(PO_4_)_2_ at 9.763 GHz (X-band, **left**) and at 34.000 GHz (Q-band, **right**), with 0.1 mT modulation amplitude. Black: experiment; color traces are simulations using spin Hamiltonian and linewidth parameters in Table 4. Blue: without HF structure; green: hyperfine structure of 3 equivalent ^135/137^Ba nuclei in their relative natural abundance; red: linear combination of the blue and green simulated spectra, in accordance with the natural abundance of Ba isotopes. The contribution of signal D is also marked in the X-band spectrum.

Figure 6 shows a zoom-in on the EPR spectra recorded at room temperature for undoped Ba_3_(PO_4_)_2_, approximately 72 h after irradiation, to minimize the interference with the D signal. The spectra are recorded with a modulation amplitude of 0.1 mT to avoid overmodulation of the sharp central line. The X- and Q-band spectra are very well reproduced by simulations using the same set of spin Hamiltonian and linewidth parameters, as shown in Table 4. The g tensor is axial, with $g_{⊥}$ slightly smaller than $g_{\|}$, both values being slightly smaller than the free-electron value g_*e*_. At this modulation amplitude, the small g-anisotropy is already resolved in the X-band spectrum, and it becomes very clear at the Q-band microwave frequency. Like signal D, the spectra exhibit a hyperfine side structure from ^135/137^Ba nuclei. An interaction with one shell of three equivalent nuclei with an axial hyperfine tensor reproduces the spectra very well. The hyperfine interaction strength is much smaller, though, than that observed in signal D, and the residual EPR linewidth is also much smaller. The linewidth for the hyperfine structure is larger than in the center of the pattern. This broadening effect is modeled in the spectra by introducing a Gaussian distribution in the hyperfine interaction strength (A-strain).

Spectra recorded with higher modulation amplitude were also simulated to make sure that no hyperfine interactions remain hidden in the noise in Figure 6. These simulations include contributions of all three signals (H^0^, signal B and signal D) and are shown in the Supporting Information, Figure S5. The reproduction of the Q-band spectra remains very good, but the agreement in the X-band is slightly worse. Our attempts to account for this additional structure failed. Probably, low-amplitude, broad additional EPR signals from other centers occur in this magnetic field region. Hence, the hyperfine interaction listed in Table 4 is the largest, and most likely the only one resolved in the room temperature B signal.

#### 2.5.2. Discussion

**Figure 7:**
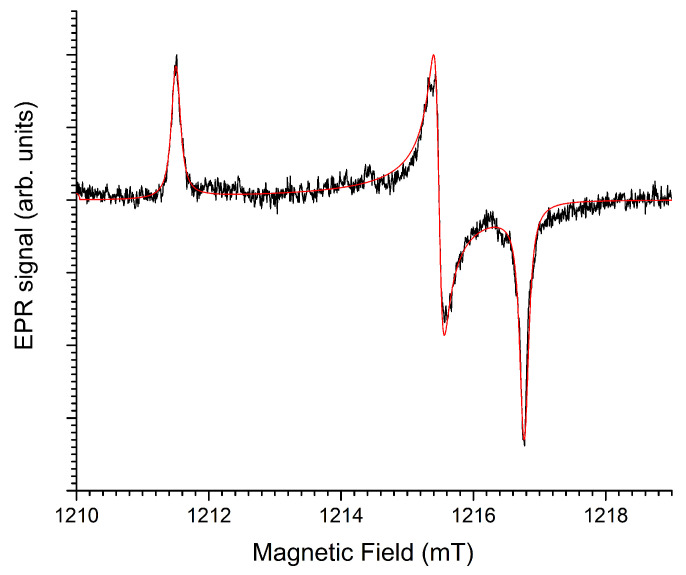
Q-band (34.000 GHz) EPR spectrum of X-irradiated undoped Ba_3_(PO_4_)_2_ at 85 K; microwave power: 32 µW; modulation amplitude: 0.1 mT. Black: experiment; red: simulation. Parameters are shown in Table 4.

Despite the similarity in symmetry and in the interaction with a shell of three Ba nuclei, the paramagnetic centers corresponding to the B and D signals show strongly different characteristics. Center B exhibits a much smaller interaction with Ba nuclei. The delocalization of the unpaired electron density onto Ba 6s orbitals of the interacting shell amounts to about 1%. The EPR linewidth only allows for very small unresolved hyperfine interactions. Contrary to center D, in center B, the unpaired electron is highly localized. In addition, the two signals strongly differ in their temperature dependence. While the D signal hardly shows any temperature dependence (Figure S4), the EPR spectrum of signal B at 85 K, shown in Figure 7, is strongly different from that at room temperature. The g tensor symmetry is reduced to rhombic, and the anisotropy ($g_{\max}$–$g_{\min}$) has strongly increased, while the average g value has hardly changed. Thus, the axial symmetry observed at room temperature does not reflect the true symmetry of the paramagnetic center, but is the result of motional averaging, similar to that observed for certain CO_2_^−^ radicals in hydroxyapatite, e.g., as in [31]. The low temperature principal g-factors for this center show similarity with those expected for CO_2_^−^ (2.0030, 2.0015, 1.997 [31]). The principal g-factors of other paramagnetic molecular anion defects, like SO_3_^−^ in K_2_S_2_O_6_ [32], are also in the range of those we observe for center B, though. Thus, based on the g tensor characteristics alone, we cannot conclusively identify this center, although it seems anion impurity-related. It is, however, not clear why such impurities would reproducibly be grown into Ba_3_(PO_4_)_2_ synthesized under diverse conditions, including in-house synthesis, commercial production and synthesis in another lab several decades ago. Based on evidence available at this moment, we tentatively assign signal B to a molecular anion defect, possibly impurity-related, located at a site in the lattice where it can reorient at room temperature so that its average symmetry is axial.

## 3. Materials and Methods

### 3.1. Sample Preparation and Characterization

Ba_2.99_Eu_0.01_(PO_4_)_2_ samples were synthesized via solid-state reaction, as detailed in Ref. [15]. BaCO_3_ (Alfa Aesar, Haverhill, MA, USA, 99.8%), (NH_4_)_2_HPO_4_ (Sigma Aldrich, St. Louis, MO, USA, 98%) and Eu_2_O_3_ (Alfa Aesar, 99.99%) were weighed in stoichiometric amounts and thoroughly mixed. Eu^3+^-doped Ba_3_(PO_4_)_2_ was obtained by calcining the mixed products for 5 h at 950 °C in air, followed by 5 h sintering at 1300 °C in air. For Eu^2+^-doping the sample was calcined for 5 h at 300 °C in air and then sintered for 5 h at 1300 °C in a 90% N_2_+10% H_2_ atmosphere. Undoped Ba_3_(PO_4_)_2_ was purchased (99.9% Sigma Aldrich). The phase purity of the materials was checked with X-ray diffraction (Bruker-Siemens D5000 and Bruker D8 Advance, Karlsruhe, Germany) using Cu-Kα radiation. The main peaks in the diffraction patterns (Supporting Information, Figure S2) match those for the desired R$\bar{3}$m trigonal phase of Ba_3_(PO_4_)_2_ (Crystallography Open Database, entry 1544726 (1544726.cif) [1]). The remaining weak diffraction peaks in the pattern of commercial Ba_3_(PO_4_)_2_ could all be attributed to Ba_5_(PO_4_)_3_(OH) [24]. No indications of other phases were found. Visualization of the unit cell of Ba_3_(PO_4_)_2_ (Figure 1) and of the defect model for center D (Figure 5) are made with the aid of VESTA [33].

### 3.2. Irradiation

X-ray irradiation was performed with unfiltered radiation of a Cu-anode X-ray tube (Siemens KFL Cu 2KDC) operated at 40 kV and 40 mA in a Siemens D5000 X-ray diffractometer (Munich, Germany). The air kerma dose rate on the sample position was estimated at 0.25 Gy/s, using the manufacturer’s specifications of the X-ray source together with the geometric configuration of the setup, including the distance and angle between the copper target and the sample. Because no additional filtering or collimation of the X-ray beam was applied, the estimate is considered reliable, but the uncertainty cannot be quantified. Samples for EPR investigation were typically irradiated for 15–20 min (225–300 Gy). Varying irradiation conditions yielded comparable relative contributions of signals A, B and D in the EPR spectra.

### 3.3. RPL Measurements

The RPL measurements were performed by exposing a previously unirradiated Eu^3+^-doped Ba_3_(PO_4_)_2_ sample for short intervals to X-rays, and subsequently measuring the PL while blocking the X-rays with Pb shutters. A 375 nm high power LED was used for simultaneous excitation of the Eu^2+^ and Eu^3+^ PL emitted by the sample, which was captured by a 1 mm diameter optical fiber guided to an AvaSpec-Hero spectrometer (Avantes, Apeldoorn, The Netherlands). The PL measurements were executed with a short integration time (2 s) to minimize possible effects of the excitation light on the defect charge states.

### 3.4. EPR Measurements

Q-band EPR experiments were conducted using a Bruker ElexSys E500 spectrometer (Bruker BioSpin GmbH, Rheinstetten, Germany) operating at the Q-band (34 GHz), with an ER 5106 QT-W resonator and equipped with a Pendulum CNT-90XL frequency counter (Pendulum Instruments AB, Stockholm, Sweden). Magnetic fields were calibrated, using the spectrum of an isotropic CO_2_^−^ signal in hydroxyapatite (g=2.0004) as a reference [31,34]. Most of the experiments presented in this paper were recorded at room temperature. For the measurements performed at 85 K, the sample was cooled using liquid N_2_ in an Oxford CF935 gas flow cryostat (Oxford Instruments, Abingdon, UK). X-band EPR measurements were all performed at room temperature using a Bruker ESP300E spectrometer with a standard (ER 4102ST) or a high-quality EPR cavity (4122SHQE/0401), equipped with a HP 5350B microwave frequency counter (Hewlett-Packard, Palo Alto, CA, USA). Magnetic fields were calibrated against the spectrum of diphenyl picrylhydrazyl (DPPH, g=2.0036). Both in X- and in Q-band spectra, 100 kHz field modulation was applied with an amplitude of 0.1–0.5 mT.

### 3.5. EPR Spectrum Simulations

EPR spectra were simulated by diagonalization of the appropriate spin Hamiltonian using the EasySpin libraries [29] in MATLAB R2021b (The MathWorks Inc., Natick, MA, USA). Linewidths presented in Table 1, Table 2, Table 3 and Table 4 are peak-to-peak values (lwpp). Ba has two stable magnetic isotopes: ^135^Ba (I = 3/2, $g_{N}$ = 0.55863, natural abundance 6.592%) and ^137^Ba (I = 3/2, $g_{N}$ = 0.62491, natural abundance 11.232%). The other natural stable isotopes of Ba have I = 0. For the simulation of the hyperfine structure with Ba nuclei, only centers without and with one magnetic nucleus in the immediate environment were taken into account. The contribution of centers interacting with more than one magnetic nucleus was neglected. The most important reason for this approximation is that EasySpin simulations only allow strain in one hyperfine interaction. Figure S6 in the Supporting Information shows a comparison between simulations using this approach and simulations including all isotopic combinations for centers B and D (for labeling, see further in the text), without broadening by strain in the hyperfine values. This comparison reveals only very subtle effects of centers interacting with more than one magnetic Ba nucleus, justifying our approach.

## 4. Conclusions

Our EPR results demonstrate that stable trapping of radiation-induced electrons by Eu^3+^ in Ba_3_(PO_4_)_2_ occurs preferentially at one specific Ba^2+^ site in the lattice. In Eu^3+^-doped Ba_3_(PO_4_)_2_ this is by far the most abundant stable electron trap, detectable with EPR at room temperature. Electrons or holes trapped at other intrinsic or accidental impurity-related defects also contribute to the room temperature EPR spectrum. These include H^0^ species characterized by a hyperfine splitting of ∼50 mT (labeled A in Ref. [2]), an oxygen-vacancy-related center (labeled D in [2], with g∼1.976), and B centers with an average *g*-factor very close to 2.0000, which probably are related to an accidental anion impurity. Several broad EPR components with g>2.0023 remain unidentified, which are produced in much larger concentrations in Eu^3+^-doped Ba_3_(PO_4_)_2_, and are therefore most likely trapped hole centers.

Understanding the site selectivity for electron trapping by Eu^3+^ ions and the hole trap defect chemistry is essential for tailoring Ba_3_(PO_4_)_2_:Eu^3+^ as an efficient RPL phosphor. Our future work will therefore focus on determining the incorporation sites of RE^3+^-doped Ba_3_(PO_4_)_2_, combining optical and magnetic resonance spectroscopy, identifying the trapped hole centers, and engineering trapping in this RPL phosphor by co-doping. 

## Figures and Tables

**Table 1:** Spin Hamiltonian and linewidth parameters used in the simulation of the Eu^2+^ EPR spectra in Figure 1. *g* and $B_{k}^{q}$ values are reproduced from [15]. No error is given for the linewidth parameter. Its value should only be regarded as indicative: it is chosen in such a way to account for the hyperfine interaction with the Eu nucleus, which is only partly resolved in some transitions.

Eu^2+^ Center	Site 1	Site 2
*g*	1.992±0.001	1.992±0.001
$B_{2}^{0}$ (MHz)	1300±10	680±10
$B_{4}^{0}$ (MHz)	0.25±0.10	0.35±0.10
Linewidth (mT, Lorentzian)	8	8

**Table 2:** Spin Hamiltonian parameters for the H^0^ center in Ba_3_(PO_4_)_2_.

H^0^	This Paper	Ref. [2]
*g*	2.0024±0.0002	2.013
A (mT)	49.7±0.2	50.3
Linewidth (mT, Lorentzian)	0.12±0.03	n.a.

**Table 4:** Spin Hamiltonian and linewidth parameters for signal B.

Signal B	Parameter	Value ± Error
Room temperature	$g_{⊥}$	2.0012±0.0002
	$g_{\|}$	1.9986±0.0002
	$A_{⊥}$ (mT)	0.65±0.02
	$A_{\|}$ (mT)	0.78±0.02
	A strain (mT)	0.18±0.02
	Linewidth (Lorentzian, mT)	0.16±0.02
85 K	$g_{x}$	2.0051±0.0002
	$g_{y}$	1.9986±0.0002
	$g_{z}$	1.9965±0.0002
	Linewidth (Lorentzian, mT)	0.08±0.02

## Data Availability

The raw data supporting the conclusions of this article will be made available by the authors upon request.

## Supplementary Materials

The following supporting information can be downloaded at: https://www.mdpi.com/article/10.3390/molecules31061045/s1. Additional structural and EPR characterization data are provided in the Supporting Information, including X-ray diffraction patterns of undoped and Eu-doped Ba_3_(PO_4_)_2_ (Figure S2), Q-band EPR spectra after UV and X-ray irradiation (Figures S1 and S3–S5), and simulations comparing different hyperfine interaction models involving magnetic Ba nuclei (Figure S6).
