# Supplementary material for: Electron Paramagnetic Resonance Study of Radiation-Induced Defects in Ba3(PO4)2
Source: Molecules. 2026 Mar 20;31(6):1045. doi: 10.3390/molecules31061045 (PMC13029406; doi:10.3390/molecules31061045)
Supplement: Supplementary file 1 [file molecules-31-01045-s001.zip › molecules-4198761-supplementary.pdf]

Article

Supporting Information: Electron Paramagnetic Resonance Study of Radiation-Induced Defects in $Ba_3(PO_4)_2$

Henk Vrielinck [1]*, Wouter Holvoet [1], Dominykas Augulis [1,2], Eliot Janssens [1], David Van der Heggen [1] and Dirk Poelman [1,]

[1] Ghent University, Department of Solid State Sciences, Krijgslaan 285 - S1, B-9000 Gent, Belgium
[2] Institute of Photonics and Nanotechnology, Faculty of Physics, Vilnius University, Sauletekio al. 3, LT-10257 Vilnius, Lithuania
* Correspondence: henk.vrielinck@ugent.be

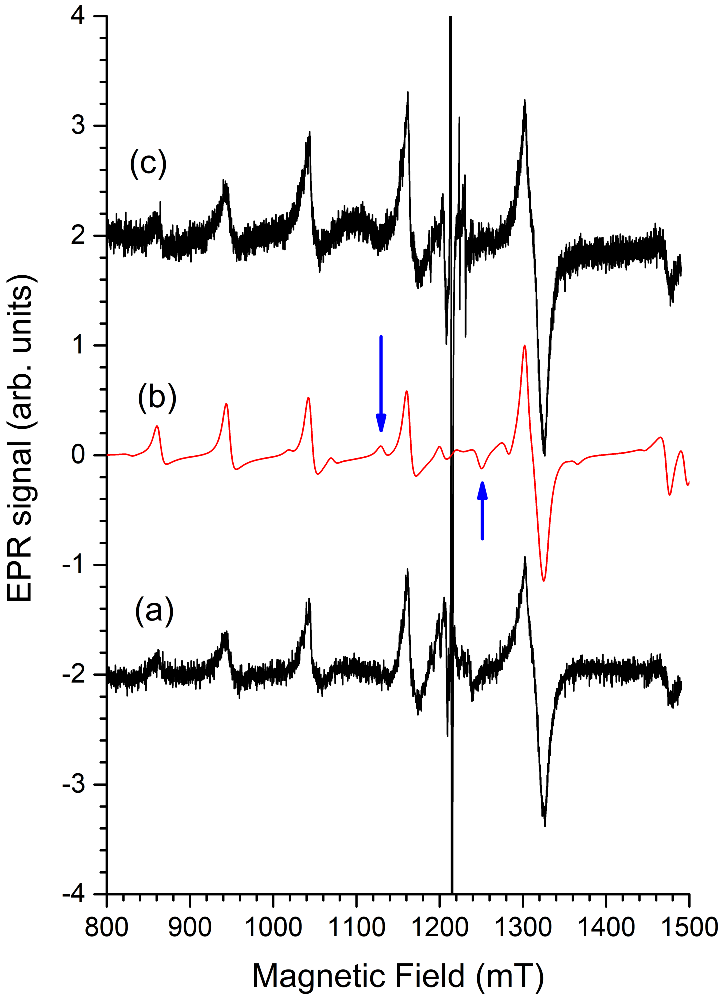

Figure S1. Room temperature EPR spectrum of $Ba_3(PO_4)_2$:Eu^{3+} after irradiation: a) with UV (λ = 265 nm, $\sim$ 1J/cm^{-2}) ; b) simulation assuming an intensity ratio for Eu^{2+} centers Site 1 and Site 2 of 1:8 ; c) with X-rays (40 kV, 40 mA, $\sim$225 Gy). Green arrows indicate features of Eu^{2+} center Site 1 in the simulation that are clearly missing in the experimental spectra.

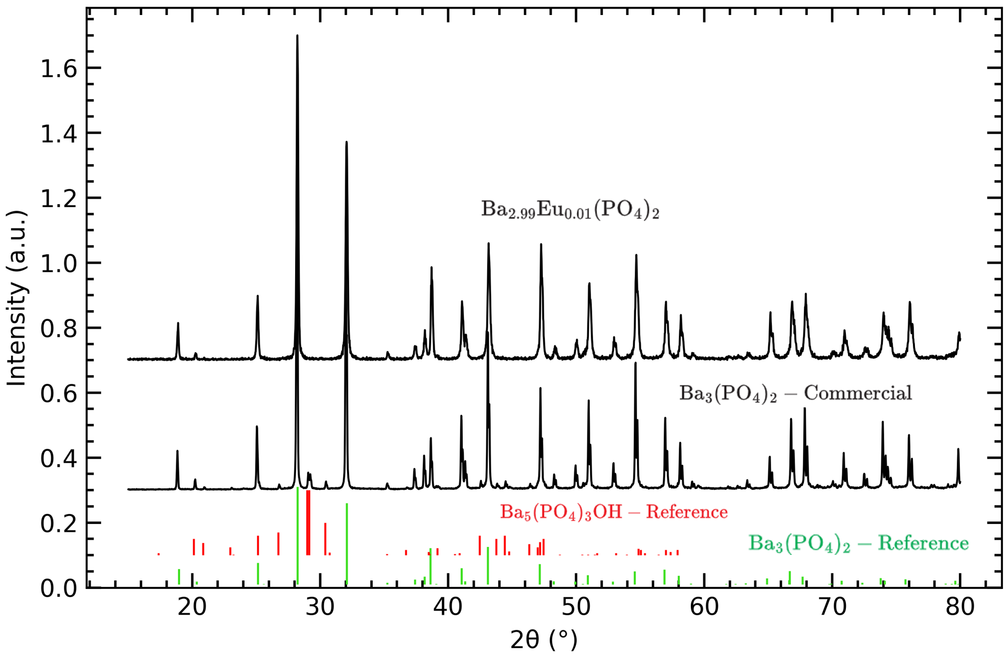

Figure S2. X-ray diffractogram measured on the commercial $Ba_3(PO_4)_2$ sample and Eu doped sample. All high-intensity diffraction peaks are due to the $Ba_3(PO_4)_2$ phase. $Ba_5(PO_4)_3OH$ — Reference XRD pattern calculated from the structural data reported in [1]. $Ba_3(PO_4)_2$ — Reference structure from the Crystallography Open Database (entry 1544726, [2]).

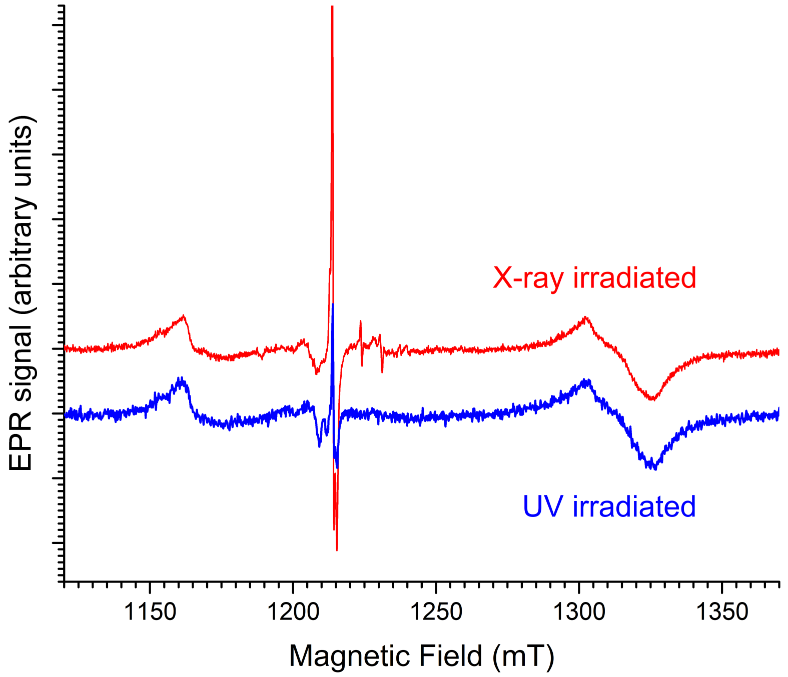

Figure S3. Q-band EPR spectrum of UV charged (λ = 265 nm, $\sim$ 1 J/cm^2) and X-ray irradiated Eu-doped $Ba_3(PO_4)_2$. Room temperature spectra, normalized on the signal height of the Eu^{2+} signal.

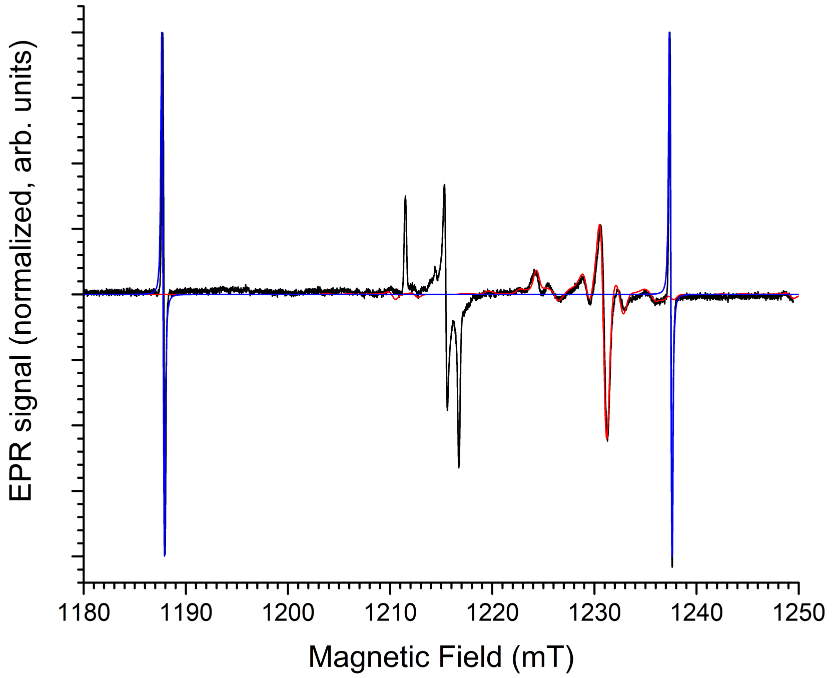

Figure S4. 85 K Q-band EPR spectrum (34.000 GHz) of undoped $Ba_3(PO_4)_2$ after X-ray irradiation at room temperature (50 kV, 40 mA, 1800 s). Microwave power: 0.127 mW ; modulation amplitude: 0.3 mT. Black trace – experimental; Red trace – simulation with the spin Hamiltonian parameters for Signal D, determined from room temperature spectra (Table 3, main text). Blue trace - simulation with the spin Hamiltonian parameters for H0, determined from room temperature spectra (Table 2, main text). The structure of the EPR spectrum for these two paramagnetic centers hardly depends on temperature.

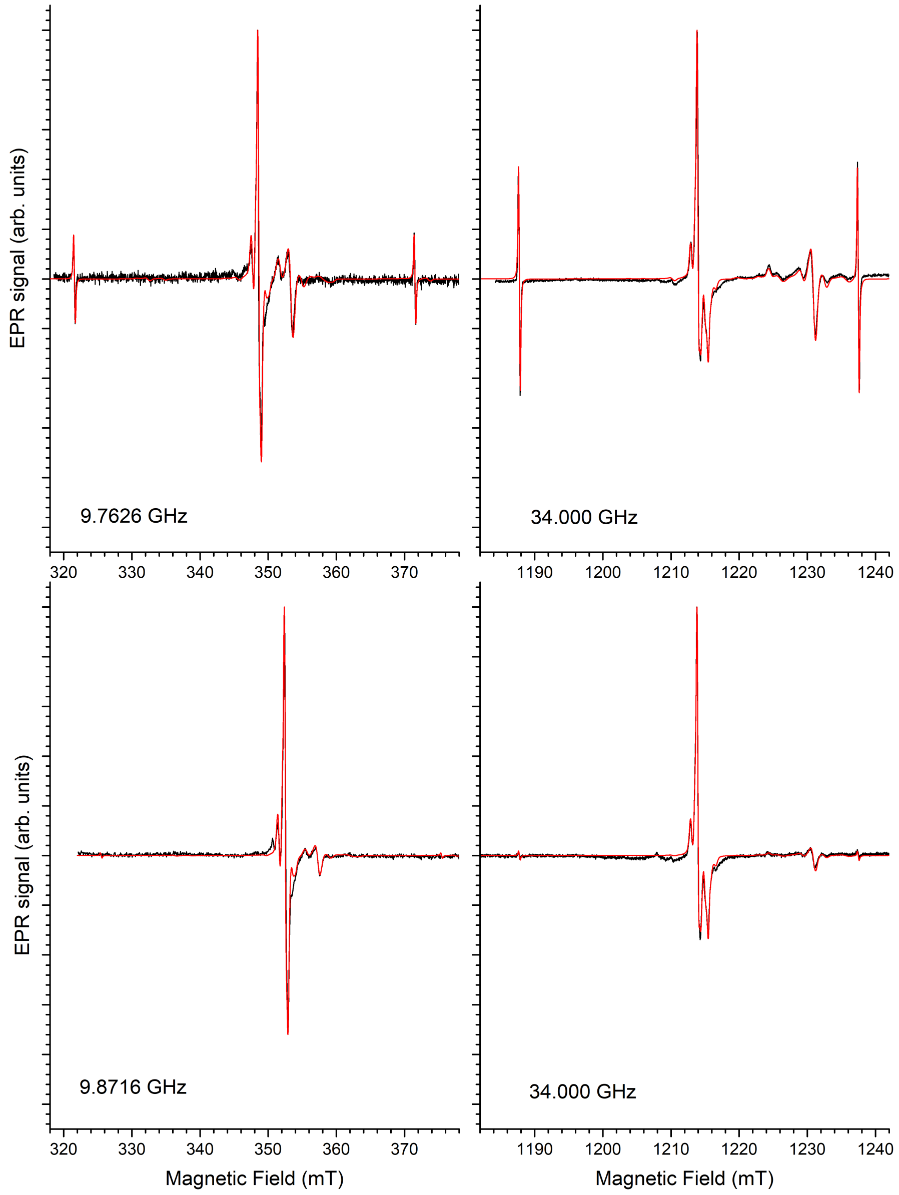

Figure S5. Room temperature EPR spectra of X-ray irradiated undoped $Ba_3(PO_4)_2$, recorded with 0.3 mT modulation amplitude, to improve signal-to-noise ratio, attempting to highlight the hyperfine structure on the B signal, recorded ∼ 2 h and ∼ 72 h after irradiation in X- and Q-band

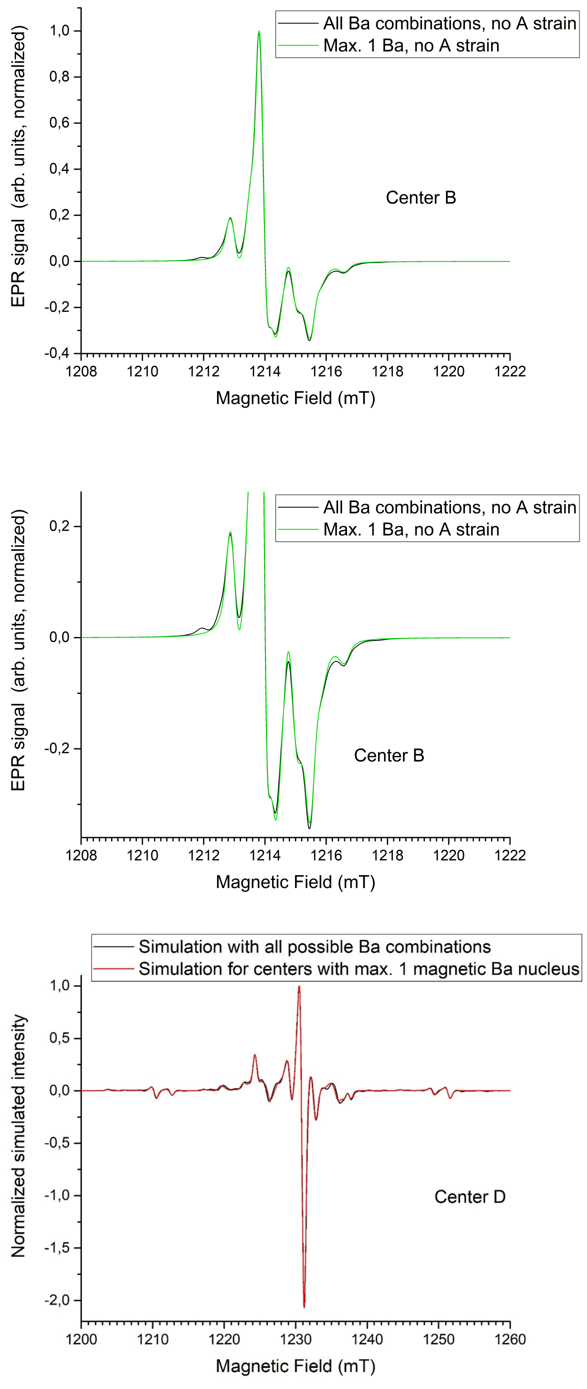

Figure S6. Comparison between simulations with all possible combinations of magnetic Ba nuclei (black) and assuming that only one magnetic Ba nucleus is within set of Ba nuclei for which hyperfine interaction is observed (colored trace).

1. Duan, C.J.; Wu, X.Y.; Liu, W.; Chen, H.H.; Yang, X.X.; Zhao, J.T. X-ray excited luminescent properties of apatitic compounds $Ba_5(PO_4)_3X$ (X: OH^-, Cl^-, Br^-); structure and hydroxyl ion conductivity of barium hydroxylapatite. *Journal of Alloys and Compounds* **2005**, *396*, 86–91. https://doi.org/https://doi.org/10.1016/j.jallcom.2004.11.064.
2. Sugiyama, K.; Tokonami, M. The crystal structure refinements of the strontium and barium orthophosphates. *Mineral. J.* **1990**, *15*, 141–146. https://doi.org/10.2465/minerj.15.141.
